# DAT-SPECT-based subtype and stage inference in Parkinson’s disease

**DOI:** 10.1038/s41531-026-01347-2

**Published:** 2026-04-15

**Authors:** Masakazu Ozawa, Daisuke Yoshimaru, Ami Yuzawa, Kenji Ishii, Hirotaka James Okano

**Affiliations:** 1 Division of Regenerative Medicine, The Jikei University School of Medicine, Tokyo, Japan; 2 Research Team for Neuroimaging, Tokyo Metropolitan Institute for Geriatrics and Gerontology, Tokyo, Japan; 3 Department of Neurology, The Jikei University School of Medicine, Tokyo, Japan

**Keywords:** Biomarkers, Diseases, Medical research, Neurology, Neuroscience

## Abstract

Heterogeneity in the pattern of nigrostriatal degeneration can complicate the prognosis and treatment of Parkinson’s disease. We analysed dopamine transporter single-photon emission computed tomography with ^123^I-ioflupane (DAT-SPECT) using atlas-based 12-region segmentation in 636 drug-naive patients with sporadic Parkinson’s disease and 126 healthy controls. Subtype and Stage Inference (SuStaIn) algorithm was applied to multi-regional DAT-SPECT data at baseline, 1, 2, and 4 years. SuStaIn identified three reproducible subtypes of nigrostriatal dopaminergic degeneration: (S1) left posterior putamen, (S2) right posterior putamen, and (S3) bilateral caudate, with high longitudinal stability. At baseline, S3 patients were older, exhibited greater cognitive and psychiatric burden, and demonstrated lower cerebrospinal fluid α-synuclein seeding positivity. Longitudinally, motor symptoms improved more in S1 and S2 than in S3 upon initiation of treatment, whereas depression and anxiety worsened preferentially in S2 and impulsive–compulsive behaviours increased in S3. DAT-SPECT-based SuStaIn thus reconstructs biologically plausible dopaminergic progression patterns and yields clinically meaningful subtypes with short-term prognostic relevance.

## Introduction

Nigrostriatal dopaminergic denervation is a hallmark of Parkinson’s disease, and its severity is closely related to lateralized motor symptoms and disturbances of cognition, mood, sleep, and autonomic function^1–3^. However, the location and rate of degeneration substantially varies across individuals, likely contributing to differences in disease presentation, treatment response, and prognosis^4^. Consequently, objective biomarkers that can capture this heterogeneity are required for precise phenotyping and clinical care. Dopamine transporter single-photon emission computed tomography with ^123^I-ioflupane (DAT-SPECT) assesses the presynaptic dopamine transporter (DAT) availability as a proxy for nigrostriatal dopaminergic degeneration and is widely employed in Parkinson’s disease diagnosis^5^. Because dopaminergic medications substantially influence clinical symptoms, baseline clinical assessments are preferably performed in drug-naive conditions^5,6^. A posterior-to-anterior gradient with early, often asymmetric, posterior putaminal involvement and relatively early sparing of the caudate has been previously reported^7^. Additionally, subgroup observations, including comparatively early caudate involvement and distinct lateralization, have suggested multiple routes of progression^8^.

Data-driven disease modelling provides a complementary approach to examining these trajectories. The Subtype and Stage Inference (SuStaIn) framework reconstructs distinct progression sequences and assigns each individual a most-likely subtype and stage based on cross-sectional data, distinguishing phenotypic from temporal heterogeneity^9^. To our knowledge, SuStaIn has not been systematically applied to atlas-based, multiregional DAT-SPECT data in Parkinson’s disease. Leveraging a more detailed striatal parcellation could yield biologically plausible progression patterns and enable robust, site-independent staging.

Using a large, multi-site, drug-naive cohort with harmonized 12-region DAT-SPECT data, we aimed to: (1) derive SuStaIn-based subtypes and stages of nigrostriatal dopaminergic degeneration inferred from regional DAT availability and assess their cross-sectional plausibility; (2) evaluate longitudinal stability at approximately 1-, 2-, and 4-year follow-ups; and (3) examine clinical relevance by relating subtype and stage to baseline phenotype as well as to prospective motor and psychiatric trajectories under routine care.

## Results

### Cohort, SuStaIn subtypes, and longitudinal stability

**Fig. 1:**
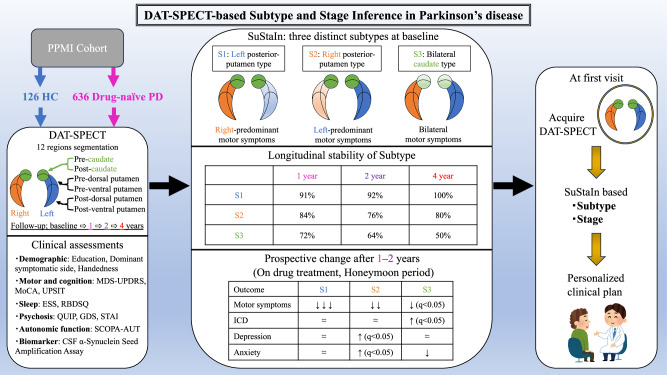
Graphical overview of DAT-SPECT-based subtype and stage inference in Parkinson’s disease. Overview of the study pipeline and clinical use case. Left: PPMI cohort and atlas-based DAT SPECT regional quantification (12-region striatal parcellation). Middle-top: SuStaIn identified three subtypes—S1: left posterior-putamen type; S2: right posterior-putamen type; S3: bilateral caudate type—with schematic distribution of regional loss. Middle-center: longitudinal validation of subtype assignments shown as transition at 1, 2, and 4 years (row percentages). Middle-bottom: prospective change after 1–2 years (on treatment); arrows denote direction ( ↓ improvement; ↑ worsening; ≈ no change), with Benjamini–Hochberg–adjusted *q* < 0.05 applied within each outcome. Right: conceptual clinical workflow illustrating how DAT-SPECT–based SuStaIn analysis may support individualized subtype and stage assignment and inform risk stratification and follow-up strategies.

**Fig. 2:**
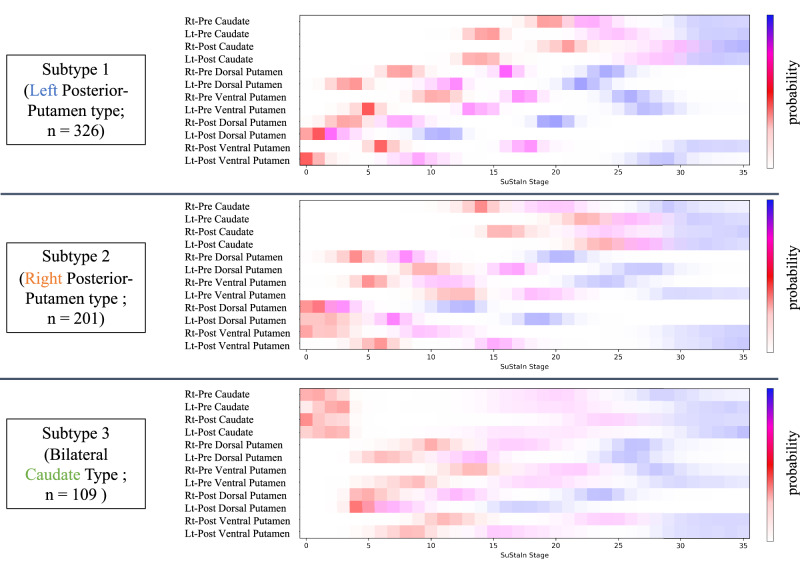
SuStaIn-derived progression patterns across 12 DAT-SPECT regions. We incorporated DAT-SPECT measures from 12 striatal subregions and included age, sex, site, and handedness as covariates. The heat maps show a unidirectional progression from warm to cold as increasing stage (warm → cold indicates worsening, i.e., greater DAT deficit).

**Fig. 3:**
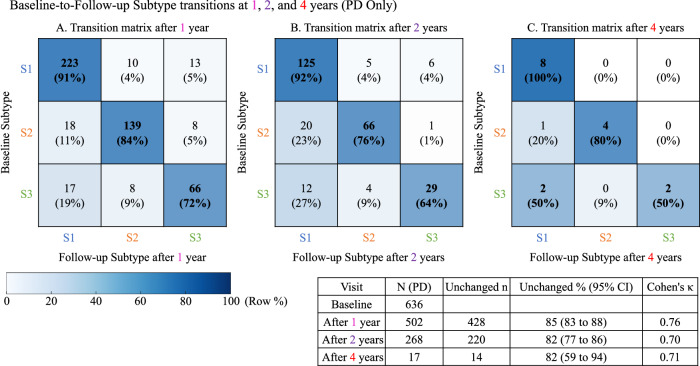
Baseline-to-follow-up subtype transitions at 1, 2, and 4 years (PD only). Row-normalized transition matrices from baseline subtype (rows) to follow-up subtype (columns) at: **A** 1 year, **B** 2 years, **C** 4 years. Cells show counts and row percentages; color encodes stability (darker = higher). Inset table reports *n* per visit, unchanged proportion with 95% CI, and Cohen’s *κ* as a measure of agreement across time. Note the small sample size at 4 years.

The design of this study (Fig. 1) includes the analysis of the analytical cohort, SuStaIn-derived subtypes, their longitudinal stability, and the potential clinical workflow. The analytical cohort consisted of drug-naive patients with sporadic Parkinson’s disease (n = 636) and healthy controls (HC; *n* = 126), all imaged using DAT-SPECT (Supplementary Fig. 1). Clinical variables were available for most participants in both groups, with sporadic missingness for select measures (e.g. cerebrospinal fluid α–synuclein seed amplification assay (CSF-SAA)). Exact sample sizes for each analysis are provided in Table 1. SuStaIn identified three distinct subtypes of nigrostriatal dopaminergic degeneration defined by their initial regional involvement: (i) left posterior putamen (S1; n = 326), (ii) right posterior putamen (S2; *n* = 201), and (iii) bilateral caudate (S3; n = 109) (Fig. 2). Application of the baseline-trained model to follow-up scans demonstrated high temporal stability of subtype assignments (Fig. 3). The proportion of participants remaining within the same subtype across ~1-, 2-, and 4-year follow-ups was as follows: S1, 91% → 92% → 100%; S2, 84% → 76% → 80%; and S3, 72% → 64% → 50%. Despite smaller sample sizes at year 4, the overall subtype retention remained robust (85% → 82% → 82%), with Cohen’s κ values of approximately 0.76, 0.70, and 0.71, respectively.

**Table 1:** Participant characteristics and group comparisons

	Healthy Control (HC)		Subtype 1 (S1)		Subtype 2 (S2)		Subtype 3 (S3)		4 group comparison (HC vs S1 vs S2 vs S3)		3 group comparison (S1 vs S2 vs S3)	
			Lt Posterior-Putamen type		Rt Posterior-Putamen Type		Bilateral Caudate Type		Kruskal–Wallis	Post-hoc Bonferroni	Kruskal–Wallis	Post-hoc Bonferroni
	n = 126	mean ± SD	n = 326	mean ± SD	n = 201	mean ± SD	n = 109	mean ± SD	*P* value	*Q* value < 0.05	*P* value	*Q* value < 0.05
Stage	n = 126	1.5 ± 2.5	n = 326	15.1 ± 7.4	n = 201	15.0 ± 6.8	n = 109	15.3 ± 7.3	<0.001***	HC < S1, S2, S3	0.882	
Number of sites	n = 126	33	n = 326	47	n = 201	43	n = 109	39				
Number of patients per site	n = 126	3.8 ± 2.5	n = 326	6.9 ± 4.2	n = 201	4.7 ± 3.1	n = 109	2.8 ± 2.4	<0.001***	HC, S3 < S2; S2, S3 < S1	<0.001***	S3 < S2; S2, S3 < S1
Sex (female: male)	n = 126	65: 61	n = 326	122: 204	n = 201	86: 115	n = 109	12: 97	a) <0.001***		a) <0.001***	
Age (years)	n = 126	64.3 ± 11.5	n = 326	64.1 ± 9.5	n = 201	62.9 ± 8.9	n = 109	66.6 ± 8.5	0.003**	S2 < HC, S3	0.002**	S1, S2 < S3
Education (years)	n = 126	16.4 ± 2.8	n = 325	16.2 ± 2.7	n = 200	16.3 ± 2.8	n = 109	16.5 ± 2.7	0.499		0.412	
Disease duration (month)	n = 0		n = 326	10.5 ± 7.6	n = 201	9.9 ± 6.9	n = 109	10.0 ± 7.4	0.733		0.733	
LED (mg)	n = 126	0	n = 326	0	n = 201	0	n = 109	0				
BMI (kg/cm^2^)	n = 126	26.5 ± 4.8	n = 322	26.7 ± 5.2	n = 198	26.6 ± 5.4	n = 107	27.0 ± 3.6	0.400		0.293	
Handedness (Rt: Lt: Bilateral)	n = 126	114: 11: 1	n = 326	301: 18: 7	n = 201	174: 19: 8	n = 109	85: 18: 6	a) 0.003**	HC, S1 < S3	a) 0.002**	
DomSymSide (Rt: Lt: Bilateral)	n = 0		n = 322	271: 50: 1	n = 200	12: 186: 2	n = 105	63: 39: 3			a) <0.001***	
Tremor/PIGD ratio	n = 126	1.8 ± 0.4	n = 324	1.3 ± 0.4	n = 201	1.3 ± 0.4	n = 109	1.4 ± 0.5	<0.001***	S1, S2, S3 < HC	0.114	
MDS-UPDRS Part I	n = 126	3.9 ± 3.3	n = 325	6.4 ± 4.5	n = 197	6.3 ± 4.6	n = 107	8.0 ± 5.2	<0.001***	HC < S1, S2, S3; S1, S2 < S3	0.005**	S1, S2 < S3
MDS-UPDRS Part II	n = 126	0.5 ± 1.0	n = 325	6.5 ± 4.7	n = 201	5.9 ± 4.2	n = 109	7.5 ± 5.0	<0.001***	HC < S1, S2, S3	0.012*	S2 < S3
MDS-UPDRS Part III	n = 124	2.0 ± 2.4	n = 323	22.4 ± 9.6	n = 201	23.4 ± 10.5	n = 107	23.0 ± 9.9	<0.001***	HC < S1, S2, S3	0.549	
UPSIT	n = 122	33.2 ± 6.1	n = 313	23.0 ± 7.7	n = 191	23.0 ± 7.5	n = 106	21.9 ± 7.9	<0.001***	S1, S2, S3 < HC	0.365	
MoCA	n = 126	27.4 ± 2.1	n = 324	26.7 ± 2.6	n = 199	27.2 ± 2.1	n = 109	26.6 ± 2.5	0.006**	S3 < HC	0.034*	S3 < S2
QUIP	n = 126	0.2 ± 0.5	n = 324	0.2 ± 0.6	n = 201	0.2 ± 0.5	n = 109	0.3 ± 0.5	0.154		0.158	
ESS	n = 126	4.5 ± 3.1	n = 326	5.3 ± 3.6	n = 200	5.4 ± 4.0	n = 108	6.3 ± 3.8	0.002**	HC < S3	0.029*	S1 < S3
RBDSQ	n = 126	2.3 ± 2.6	n = 325	3.9 ± 3.0	n = 200	4.0 ± 3.2	n = 109	4.3 ± 3.1	<0.001***	HC < S1, S2, S3	0.517	
GDS	n = 126	1.4 ± 2.4	n = 325	2.1 ± 2.5	n = 200	2.0 ± 2.5	n = 107	3.2 ± 3.2	<0.001***	HC < S1, S2, S3; S1, S2 < S3	0.001**	S1, S2 < S3
STAI	n = 126	53.8 ± 13.1	n = 325	62.7 ± 18.2	n = 200	61.4 ± 17.5	n = 108	66.6 ± 18.2	<0.001***	HC < S1, S2, S3; S2 < S3	0.024*	S2 < S3
SCOPA-AUT	n = 126	6.9 ± 4.8	n = 324	10.7 ± 6.8	n = 199	10.6 ± 6.7	n = 106	11.8 ± 6.9	<0.001***	HC < S1, S2, S3	0.179	
CSF-SAA, n (%)	n = 0		n = 286	264 (93.0%)	n = 182	172 (94.5%)	n = 102	84 (82.4%)			a) 0.002**	S3 < S1, S2
DAT-SPECT Rt-Pre Caudate	n = 126	1.4 ± 0.4	n = 326	1.1 ± 0.4	n = 201	1.0 ± 0.3	n = 109	0.6 ± 0.3	<0.001***	S1, S2, S3 < HC; S3 < S1, S2; S2 < S1	<0.001***	S3 < S1, S2; S2 < S1
Lt-Pre Caudate	n = 126	1.4 ± 0.4	n = 326	1.0 ± 0.3	n = 201	1.2 ± 0.4	n = 109	0.6 ± 0.3	<0.001***	S1, S3 < HC; S3 < S1, S2; S1 < S2	<0.001***	S3 < S1, S2; S1 < S2
Rt-Post Caudate	n = 126	0.5 ± 0.2	n = 326	0.4 ± 0.2	n = 201	0.4 ± 0.2	n = 109	0.2 ± 0.2	<0.001***	S1, S2, S3 < HC; S3 < S1, S2	<0.001***	S3 < S1, S2; S2 < S1
Lt-Post Caudate	n = 126	0.6 ± 0.2	n = 326	0.4 ± 0.2	n = 201	0.5 ± 0.2	n = 109	0.2 ± 0.1	<0.001***	S1, S3 < HC; S3 < S1, S2; S1 < S2	<0.001***	S3 < S1, S2; S1 < S2
Rt-Pre Dorsal Putamen	n = 126	2.1 ± 0.5	n = 326	1.3 ± 0.5	n = 201	0.9 ± 0.4	n = 109	1.2 ± 0.5	<0.001***	S1, S2, S3 < HC; S2 < S1, S3	<0.001***	S2 < S1, S3
Lt-Pre Dorsal Putamen	n = 126	2.0 ± 0.4	n = 326	1.0 ± 0.4	n = 201	1.3 ± 0.4	n = 109	1.1 ± 0.5	<0.001***	S1, S2, S3 < HC; S1, S3 < S2	<0.001***	S1, S3 < S2
Rt-Pre Ventral Putamen	n = 126	1.7 ± 0.4	n = 326	1.2 ± 0.4	n = 201	0.9 ± 0.3	n = 109	1.2 ± 0.4	<0.001***	S1, S2, S3 < HC; S2 < S1, S3	<0.001***	S2 < S1, S3
Lt-Pre Ventral Putamen	n = 126	1.7 ± 0.4	n = 326	1.0 ± 0.3	n = 201	1.2 ± 0.3	n = 109	1.1 ± 0.4	<0.001***	S1, S2, S3 < HC; S1, S3 < S2	<0.001***	S1, S3 < S2; S3 < S2
Rt-Post Dorsal Putamen	n = 126	1.4 ± 0.3	n = 326	0.6 ± 0.3	n = 201	0.4 ± 0.2	n = 109	0.6 ± 0.3	<0.001***	S1, S2, S3 < HC; S2 < S1, S3	<0.001***	S2 < S1, S3
Lt-Post Dorsal Putamen	n = 126	1.4 ± 0.3	n = 326	0.4 ± 0.2	n = 201	0.7 ± 0.2	n = 109	0.6 ± 0.3	<0.001***	S1, S2, S3 < HC; S1 < S2, S3; S3 < S2	<0.001***	S1, S3 < S2; S3 < S2
Rt-Post Ventral Putamen	n = 126	1.1 ± 0.3	n = 326	0.6 ± 0.3	n = 201	0.4 ± 0.1	n = 109	0.6 ± 0.2	<0.001***	S1, S2, S3 < HC; S2 < S1, S3	<0.001***	S2 < S1, S3; S1 < S3
Lt-Post Ventral Putamen	n = 126	1.2 ± 0.3	n = 326	0.5 ± 0.2	n = 201	0.6 ± 0.2	n = 109	0.6 ± 0.3	<0.001***	S1, S2, S3 < HC; S1, S3 < S2	<0.001***	S1, S3 < S2

Baseline demographics, clinical measures, and regional DAT-SPECT indices across healthy controls (HC) and PD SuStaIn subtypes S1–S3. Continuous variables are mean ± SD; categorical variables are counts or percentages. Group comparisons by Kruskal–Wallis (HC vs S1 vs S2 vs S3) with post-hoc pairwise tests; within-PD comparisons also shown (S1 vs S2 vs S3). *q* values denote Benjamini–Hochberg FDR across pairwise contrasts within each outcome.

*LEDD* levodopa equivalent daily dose, *BMI* body mass index, *DomSymSide* dominant symptomatic side at baseline, *PIGD* postural instability and gait difficulty, *MDS-UPDRS* movement disorder society–unified Parkinson’s disease rating scale, *MoCA* montreal cognitive assessment, *UPSIT* University of Pennsylvania Smell Identification Test, *QUIP* questionnaire for impulsive-compulsive disorders, *ESS* Epworth Sleepiness Scale, *RBDSQ* REM sleep behavior disorder screening questionnaire, *GDS* geriatric depression scale, *STAI* state–trait anxiety inventory, *SCOPA-AUT* scales for outcomes in Parkinson’s disease–autonomic dysfunction, *SBR* specific binding ratio.

### Sensitivity analysis of subtype trajectories

**Fig. 4:**
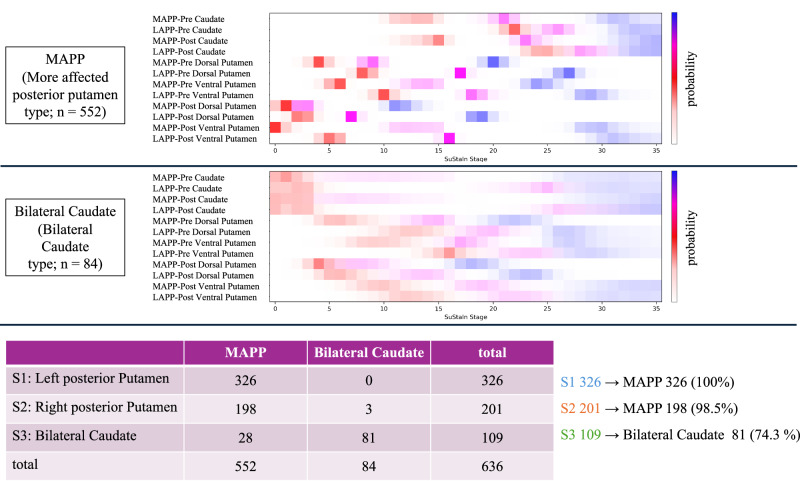
MAPP/LAPP alignment reveals robust SuStaIn subtypes in the baseline Parkinson’s disease cohort. Hemispheric involvement was aligned according to the more affected posterior putamen, defining the more affected posterior putamen (MAPP) and the less-affected posterior putamen (LAPP). Subtype progression patterns subsequently emerged as two principal trajectories originating from the MAPP and bilateral caudate. Concordance analysis revealed complete reassignment of left posterior putamen (S1) cases (326/326, 100%) and near-complete reassignment of right posterior putamen (S2) cases (198/201, 98.5%) to the MAPP type, while the majority of bilateral caudate (S3) cases remained stable (81/109, 74.3%).

MAPP/LAPP alignment resulted in two principal subtype trajectories originating from either the more affected posterior putamen (MAPP) or the bilateral caudate (Fig. 4). Notably, concordance analysis demonstrated complete reassignment of the left posterior putamen subtype (S1) to the MAPP subtype (326/326, 100%) and near-complete reassignment of the right posterior putamen subtype (S2) to the MAPP subtype (198/201, 98.5%). In contrast, most patients originally classified as the bilateral caudate subtype (S3) remained stable after alignment (81/109, 74.3%).

In a sensitivity analysis using a simplified four-region striatal parcellation, the SuStaIn model converged to a single-subtype solution (Supplementary Fig. 2). This likely reflects the limited number of regional variables available for subtype inference in the simplified model. Therefore, the finer 12-region parcellation used in the main analysis likely provided sufficient spatial resolution for the SuStaIn framework to resolve heterogeneous subtype trajectories.

### Baseline group differences and stage associations

Four-group comparisons (HC, S1, S2, and S3) revealed that patients with Parkinson’s disease had significantly worse motor and non-motor symptoms than HC, accompanied by lower DAT-SPECT specific binding ratios (SBRs) across all 12 striatal regions (Table 1). Within the Parkinson’s disease cohort, three-group analyses detected significant between-subtype differences in multiple clinical domains. The dominant symptomatic side mirrored the dopaminergic lateralization patterns (right predominance in S1; left predominance in S2). Compared with S1 and S2, patients in S3 were older and presented greater impairment on Movement Disorder Society–Unified Parkinson’s Disease Rating Scale (MDS-UPDRS) Parts I–II, lower Montreal Cognitive Assessment (MoCA) scores, and higher Epworth Sleepiness Scale (ESS), Geriatric Depression Scale (GDS), and State–Trait Anxiety Inventory (STAI) scores, along with a lower CSF-SAA positivity rate. MDS-UPDRS Part III and Questionnaire for Impulsive-Compulsive Disorders in Parkinson’s Disease (QUIP) scores at baseline did not differ significantly across subtypes. Regional DAT-SPECT findings were consistent with subtype topography, reflecting greater left putaminal loss in S1, right putaminal loss in S2, and bilateral caudate loss in S3. Exploratory linear regression models examining the association between SuStaIn stage and clinical measures (Supplementary Table 1; Supplementary Figs. 3 and 4) showed no significant relationships for HC. In S1, a higher stage was correlated with worse MDS-UPDRS Parts II–III scores and lower University of Pennsylvania Smell Identification Test (UPSIT) performance. In S2, a higher stage was associated with increased QUIP scores. In S3, a higher stage was linked to increased MDS-UPDRS Part I, STAI, and autonomic dysfunction, the Scales for Outcomes in Parkinson’s Disease–Autonomic Dysfunction (SCOPA-AUT) scores.

### Prospective longitudinal outcomes

Adjusted analysis of covariance (ANCOVA) models assessing annualized change from baseline to 1 and 2 years revealed subtype-specific trajectories (Table 2). MDS-UPDRS Part III scores improved in S1 and S2 but showed minimal improvement in S3, consistent with the early post-diagnosis ‘honeymoon period’ observed under dopaminergic therapy. Notably, psychiatric outcomes diverged over time, with QUIP scores worsening in S3, whereas GDS and STAI scores worsened in S2.

**Table 2:** Adjusted annualized changes at 1 and 2 years by subtype (primary longitudinal analysis)

Δ“Outcome 1 year” = (“Outcome 1 year”–“outcome baseline”)/“years from BL to Y1”	Subtype 1 (S1)		Subtype 2 (S2)		Subtype 3 (S3)					Pairwise (BH-adjusted)
	Lt posterior-putamen type		Rt posterior-putamen type		Bilateral caudate type					
	n = 326	EMM (95% CI)	n = 201	EMM (95%CI)	n = 109	EMM (95%CI)	F	*partial η²*	*P* value	
MDS-UPDRS Part I, 1-year annualized change	n = 269	0.52 (−0.40 to 1.44)	n = 173	0.52 (−0.45 to 1.49)	n = 94	0.81 (−0.29 to 1.91)	0.27	0.001	0.762	
MDS-UPDRS Part I, 2-year annualized change	n = 144	1.21 (0.64 to 1.78)	n = 98	1.57 (0.96 to 2.18)	n = 48	1.31 (0.57 to 2.05)	1.15	0.008	0.319	
MDS-UPDRS Part II, 1-year annualized change	n = 269	1.15 (0.15 to 2.14)	n = 177	1.09 (0.04 to 2.14)	n = 96	1.56 (0.38 to 2.75)	0.55	0.002	0.578	
MDS-UPDRS Part II, 2-year annualized change	n = 145	0.25 (−0.42 to 0.91)	n = 101	0.37 (−0.35 to 1.08)	n = 50	0.32 (−0.53 to 1.18)	0.09	0.001	0.910	
MDS-UPDRS Part III on, 1-year annualized change	n = 257	1.00 (−1.09 to 3.08)	n = 166	1.88 (−0.33 to 4.09)	n = 89	2.07 (−0.44 to 4.58)	0.96	0.004	0.385	
MDS-UPDRS Part III on, 2-year annualized change	n = 131	-2.54 (-4.11 to −0.97)	n = 98	−1.07 (-2.72 to 0.57)	n = 50	−0.42 (-2.40 to 1.56)	3.85	0.029	0.023*	S2 < S3
UPSIT, 1-year annualized change	n = 0		n = 0		n = 0					
UPSIT, 2-year annualized change	n = 0		n = 0		n = 0					
MoCA, 1-year annualized change	n = 269	0.30 (−0.24 to 0.83)	n = 173	0.27 (−0.28 to 0.83)	n = 94	−0.14 (−0.77 to 0.49)	1.84	0.007	0.159	
MoCA, 2-year annualized change	n = 143	0.03 (−0.32 to 0.39)	n = 99	−0.09 (−0.46 to 0.29)	n = 52	−0.19 (−0.64 to 0.26)	0.88	0.006	0.418	
QUIP, 1-year annualized change	n = 269	0.08 (−0.08 to 0.23)	n = 176	0.02 (−0.14 to 0.18)	n = 94	0.28 (0.10 to 0.46)	6.25	0.024	0.002**	S1, S2 < S3
QUIP, 2-year annualized change	n = 143	0.08 (−0.03 to 0.17)	n = 100	0.07 (−0.03 to 0.18)	n = 50	0.16 (0.04 to 0.29)	1.47	0.011	0.232	
ESS, 1-year annualized change	n = 271	0.18 (−0.48 to 0.85)	n = 175	0.18 (−0.53 to 0.88)	n = 95	0.73 (−0.07 to 1.52)	1.85	0.007	0.159	
ESS, 2-year annualized change	n = 147	0.37 (−0.07 to 0.81)	n = 100	0.10 (−0.38 to 0.57)	n = 51	0.61 (0.05 to 1.17)	2.23	0.016	0.110	
RBDSQ, 1-year annualized change	n = 269	0.14 (−0.39 to 0.67)	n = 175	−0.03 (−0.58 to 0.53)	n = 96	0.30 (−0.32 to 0.93)	0.89	0.003	0.413	
RBDSQ, 2-year annualized change	n = 145	0.19 (−0.20 to 0.58)	n = 101	0.33 (−0.08 to 0.75)	n = 52	0.21 (−0.28 to 0.69)	0.39	0.003	0.675	
GDS, 1-year annualized change	n = 269	0.12 (−0.37 to 0.60)	n = 174	0.19 (−0.33 to 0.69)	n = 92	0.23 (−0.36 to 0.82)	0.16	0.001	0.854	
GDS, 2-year annualized change	n = 145	0.00 (−0.30 to 0.31)	n = 99	0.33 (0.00 to 0.65)	n = 50	−0.04 (−0.43 to 0.36)	3.72	0.027	0.025*	S1 < S2
STAI, 1-year annualized change	n = 269	−0.77 (-4.06 to 2.53)	n = 175	0.80 (-2.67 to 4.27)	n = 94	−0.43 (-4.41 to 3.55)	0.86	0.003	0.425	
STAI, 2-year annualized change	n = 144	0.01 (-1.89 to 1.91)	n = 100	2.11 (0.09 to 4.12)	n = 49	−0.37 (-2.83 to 2.08)	4.19	0.030	0.016*	S1, S3 < S2
SCOPA-AUT, 1-year annualized change	n = 269	1.03 (−0.06 to 2.12)	n = 173	0.77 (−0.38 to 1.92)	n = 91	1.85 (0.54 to 3.16)	2.13	0.008	0.119	
SCOPA-AUT, 2-year annualized change	n = 146	0.28 (−0.44 to 1.00)	n = 100	0.35 (−0.42 to 1.12)	n = 50	0.51 (−0.41 to 1.43)	0.18	0.001	0.839	

Estimated marginal means (EMMs) of annualized change (95% CI) from baseline to 1 year and to 2 years across outcomes and SuStaIn subtypes. Models are ANCOVA adjusted for baseline value of the outcome, age, sex, education, site, and levodopa equivalent daily dose (LEDD) at the corresponding visit. Omnibus tests (F, P) and partial *η*² quantify subtype effects; pairwise contrasts are Benjamini–Hochberg FDR–adjusted within outcome (*q* < 0.05).

Negative values for MDS-UPDRS Part III indicate symptomatic improvement under medication (consistent with an early “honeymoon” response).

## Discussion

Herein, to our knowledge, the SuStaIn framework was applied to DAT-SPECT imaging in Parkinson’s disease for the first time. The study’s strengths include a large, drug-naive, multi-site cohort; harmonized 12-region quantitative imaging; and a prespecified, SuStaIn modelling pipeline incorporating objective stability metrics. Longitudinal analyses further extended these findings by integrating repeated DAT-SPECT and clinical assessments. In this large, drug-naive Parkinson’s Progression Markers Initiative (PPMI) cohort, the DAT-SPECT-based SuStaIn model delineated the following three subtypes: left posterior putamen (S1), right posterior putamen (S2), and bilateral caudate (S3), with high longitudinal stability across 1–4 years. Baseline comparisons confirmed the expected motor and non-motor impairments in patients with drug-naive Parkinson’s disease compared with HC, with distinct subtype-specific clinical profiles (such as older age and greater non-motor burden in S3). Longitudinally, motor symptoms improved in S1 and S2 but remained largely unchanged in S3, whereas psychiatric trajectories diverged (QUIP worsening in S3 and increased depression and anxiety scores in S2).

The findings of the present study are consistent with those reported in previous single-centre, small cross-sectional work, which identified asymmetric posterior putaminal and caudate-predominant patterns, albeit the evaluation of finer gradients was limited due to four-region segmentation (e.g. hospital-based cohorts)^10^. Additionally, studies on DAT-SPECT have shown that, at early stages, older-onset cases exhibit the posterior putamen signal comparable to that in younger-onset cases; however, the reductions in the caudate and anterior putamen are considerable, which aligns with our observation that S3 group was older at the baseline^11^. Owing to the small, single-center design, along with non-uniformity in drug-naiveness, the previous study exhibits limited generalizability. Our findings are concordant with those of previous studies and further validate the extension of observed patterns in a large, multi-center, longitudinal, drug-naive cohort with fine-grained (12-region) segmentation.

The lateralized putaminal-onset patterns observed in S1 and S2 align with the classical Parkinson’s disease asymmetry and known striatal gradient of dopaminergic degeneration. DAT-SPECT signal loss began in the posterior putamen with hemispheric asymmetry, which is consistent with prior studies^11^. In the cohort, most patients were classified as having an asymmetric posterior putamen onset (S1 or S2) at baseline, though a subset exhibited a caudate-predominant onset pattern (S3). The concordance between SuStaIn-derived subtype topography and regional SBR differences supported the biological plausibility of these subtyping outputs. During follow-up, assignments within S1 reined largely stable, whereas proportions of S2 and S3 transitioned towards S1. Collectively, these results extend prior DAT-SPECT research by introducing a fully data-driven staging and subtyping framework and demonstrating its temporal stability.

Importantly, the persistence of the bilateral caudate subtype after MAPP/LAPP alignment reduces the likelihood that this pattern merely reflects hemispheric misregistration or lateralization effects, reinforcing its biological plausibility. Together, these findings suggest that hemispheric laterality is better interpreted as the side of disease onset rather than a defining feature of subtype structure, thereby demonstrating the robustness of the identified subtype architecture.

The lower longitudinal stability observed for the caudate-onset subtype (S3) merits comment. Caudate-onset patterns have been associated with greater cognitive vulnerability previously^12^, and the S3 subtype, characterized by early bilateral caudate involvement and relatively preserved putamen, was associated with older age and greater non-motor symptom burden, a profile indicating a distinct striatal progression pattern captured by SuStaIn. Clinically, this phenotype may resemble what has been described as diffuse malignant Parkinson’s disease or, in some cases, dementia with Lewy bodies. An alternative interpretation is that S3 may be enriched for patients with atypical or mixed pathologies, given the early bilateral caudate involvement, reduced levodopa responsiveness, and lower CSF-SAA positivity. Although PPMI’s stringent diagnostic criteria and longitudinal clinical follow-up argue against a large proportion of overt non-idiopathic Parkinsonism, we cannot exclude a higher misdiagnosis rate in this subgroup. Accordingly, we view S3 as a biologically and clinically distinct dopaminergic pattern within early parkinsonism, which warrants further validation with post-mortem data and in independent cohorts.

Handedness has been linked to motor onset lateralization in Parkinson’s disease, although this uncertain association may be influenced by detection bias (e.g. heightened awareness of dominant-hand deficits)^13^. Handedness was included as a covariate, and the left posterior putamen subtype (S1) remained the most prevalent at baseline. Notably, right predominant (S2) and bilateral caudate (S3) subtypes tended to transition towards S1 over 4 years. In this predominantly right-handed cohort, we observed subtle hemispheric asymmetries in DAT-SPECT vulnerability, potentially indicating relatively greater left-sided striatal involvement. While caution is warranted, even after adjusting for handedness, the findings suggest that the putamen contralateral to the dominant hand may exhibit greater dopaminergic asymmetry, potentially contributing to the clinical impression of stronger symptoms in the dominant hand.

Although baseline linear regressions of stage on clinical measures were conducted, true prognosis requires longitudinal evaluation. Accordingly, 1- and 2-year clinical outcomes were analysed using ANCOVA, adjusting for age, sex, education, site and levodopa equivalent daily dose (LEDD), to evaluate subtype differences. At the 2-year follow-up (on-medication), improvement in MDS-UPDRS Part III followed the order S1 > S2 > S3, consistent with the typical early ‘honeymoon period’ of strong levodopa responsiveness that gradually declines over time^14,15^. In clinical practice, variability in treatment responsiveness substantially affects the quality of life and therapeutic rapport of the patient. The data suggest that DAT-SPECT-derived SuStaIn subtypes may serve as early imaging biomarkers indexing differential dopaminergic responsiveness and disease progression trajectories.

Although detailed medication class data were not considered at follow-ups, subtype-specific differences in psychiatric trajectories persisted even after adjusting for LEDD. The early ‘honeymoon period’ of Parkinson’s disease primarily reflects motor improvement, whereas non-motor symptoms often progress from the earliest disease stages, consistent with the findings of longitudinal studies^15^. Patients classified as S2 exhibited worsening depression and anxiety, reinforcing the importance of early screening and the selection of pharmacological agents with lower psychiatric adverse effects. In contrast, S3 patients demonstrated a tendency towards worsening impulsive–compulsive disorders (ICD), including behaviours such as pathological gambling and hypersexuality, which may severely disrupt patients’ and caregivers’ lives. For such patients, cautious use of dopamine agonists, which are consistently associated with a higher ICD risk (e.g. pramipexole and ropinirole) is advised^16^.

Together, these findings suggest a potentially scalable clinical framework, in which baseline DAT-SPECT acquisition combined with SuStaIn-based subtype and stage assignment may help inform risk-aware follow-up strategies. In practice, patients classified as S1 or S2 may anticipate a more favourable early motor response to dopaminergic therapy, whereas those in subtype S3 may warrant closer observation for emerging motor refractoriness and ICD. The preferential worsening of depression and anxiety in S2 highlights the need of proactive mental health screening and preventive management (e.g. counselling and judicious medication with a lower risk of psychiatric side effects). Because SuStaIn produces individualized subtype and stage estimates, it holds promise for optimising patient stratification in clinical trials and enhancing decision-support tools in routine care.

This study has some limitations, as follows: (i) the study’s observational design and non-randomized treatment exposure; (ii) follow-up attrition, particularly at the 4-year follow-up where the sample size was very small (n = 17), thereby tempering the precision of long-term estimates and introducing potential selection and attrition bias; (iii) although baseline assessments were performed in a drug-naive state and subsequent follow-up analyses adjusted for LEDD, exposure to medications that may interfere with DAT-SPECT binding (e.g. cocaine, amphetamines, methylphenidate, bupropion, and sertraline) could not be excluded, introducing potential residual confounding^6^; (iv) although all DAT-SPECT images were processed using a standardized pipeline with affine registration, precise alignment of the striatum across individuals remains technically challenging. Given the small size of the striatum, minor global registration errors and inter-individual anatomical variability (e.g., ventricular size) may affect regional correspondence, particularly in the caudate nucleus, and could contribute to apparent early caudate involvement through partial-volume or recovery effects; and (v) The limited spatial resolution of DAT-SPECT may induce correlations among neighboring subregional SBR measures; evaluating simplified regional models and validating findings in less-harmonized clinical datasets will be important to further assess robustness and generalizability. Within the scope of the current PPMI processing framework, addressing these limitations was not feasible; however, more advanced MRI-based nonlinear segmentation and partial-volume correction approaches may further improve anatomical precision in future studies.

Importantly, SuStaIn subtypes should not be interpreted as diagnostic categories, and their clinical use should complement, not replace, comprehensive clinical evaluations. Prospective validation in independent cohorts and prodromal populations with rapid eye movement sleep behaviour disorder (RBD) or hyposmia are required to test the predictive utility for disease conversion and progression. Integration with multimodal biomarkers such as meta-iodobenzylguanidine imaging, quantitative magnetic resonance imaging and plasma or CSF markers could further refine subtype biology. Methodologically, future studies incorporating longitudinal SuStaIn or hierarchical models may better capture subtype transitions and intra-individual disease patterns. Clinically, embedding SuStaIn-derived metrics into trial stratification and electronic health decision-support systems may evaluate its impact on outcomes and resource use.

Overall, the DAT-SPECT-driven SuStaIn model revealed reproducible dopaminergic progression subtypes with distinct clinical profiles and short-term prognostic relevance. Early identification of these subtypes and stages may improve the anticipation of motor response and psychiatric risks, enabling precision monitoring and individualized therapy for Parkinson’s disease.

## Methods

### Study design and participant characteristics

Data used in this study were retrieved on 9 September 2025 from the PPMI database (www.ppmi-info.org), an international, multicentre, observational study designed to identify biomarkers of Parkinson’s disease progression^17,18^. All PPMI participants provided written informed consent at enrolment, and study protocols were approved by institutional review boards at each participating site and conducted in accordance with the Declaration of Helsinki and applicable Good Clinical Practice guidelines. This research represents a secondary analysis of fully de-identified data and did not require additional institutional review board approval or assignment of a separate approval number. Ethics approval number: not applicable (secondary analysis of de-identified data). Clinical trial number: not applicable.

All PPMI participants enrolled between January 2011 and December 2024 (*n* = 4115 at baseline) were screened. From this cohort, those with genetic Parkinson’s disease (*n* = 842), prodromal RBD/hyposmia (*n* = 1,804), and scans without evidence of dopaminergic deficit (*n* = 60) were excluded, resulting in a sample of sporadic Parkinson’s disease (n = 1,080) and HC (*n* = 329). To maintain a drug-naive cohort, Parkinson’s disease cases with a non-zero LEDD were excluded at baseline (*n* = 16). Atlas-based 12-region DAT-SPECT parcellation was unavailable for early PPMI scans and was implemented between October 2020 and November 2024. After excluding cases with missing quantitative data (Parkinson’s disease, *n* = 418; HC, *n* = 193), scans acquired with 99mTc-TRODAT-1 (Parkinson’s disease, *n* = 10; HC, *n* = 10) were also excluded to ensure radiotracer consistency. Hence, the final analytical sample comprised drug-naive patients with Parkinson’s disease (*n* = 636) and HC (*n* = 126), with all presenting with complete quantitative measurements and imaged with ^123^I-ioflupane SPECT (Supplementary Fig. 1). All clinical assessments and DAT-SPECT data were obtained at the baseline levels, with follow-up assessments at ~1 year (n = 502), 2 years (*n* = 268), and 4-years (*n* = 17) when available. Minor missingness in individual clinical variables is reported as exact numbers in Table 1.

### Clinical assessments

At each visit, sex, age, years of education, disease duration, LEDD, body mass index, and handedness of participants were recorded. The site represents the PPMI imaging/recruiting center for DAT-SPECT, with the number of sites indicating the count of distinct centers contributing to the analytic cohort. The number of patients per site is reported as the mean across centers. Motor symptoms were assessed longitudinally based on the MDS-UPDRS^19^ Part III (“ON” status), with a negative change denoting symptomatic improvement. The dominant symptomatic side was determined from lateralized MDS-UPDRS Part III hemibody totals (right versus left); the side with the higher score was classified as dominant, and ‘Bilateral’ was assigned when totals were equal. The tremor/postural instability and gait difficulty (PIGD) ratio was computed by dividing the tremor score by the PIGD score using standard MDS-UPDRS item sets. Participants were classified as tremor-dominant ( ≥ 1.15), PIGD-dominant ( ≤ 0.90), and mixed/indeterminate (0.90–1.15)^20^.

Different indices were assessed as follows: global cognition, the MoCA^21^; olfaction, the UPSIT^22^; ICD, the QUIP^23^; depression, the GDS^24^; anxiety, the STAI^25^; daytime sleepiness, the ESS^26^; RBD, the RBD Screening Questionnaire (RBDSQ)^27^; and autonomic dysfunction, the SCOPA-AUT^28^. Additionally, the CSF-SAA was evaluated as described in PPMI procedures. All the results were categorized as positive, negative, or indeterminate based on pre-specified kinetic criteria (lag time/maximum fluorescence)^18^.

### DAT-SPECT acquisition, reconstruction, and segmentation

The image data used in this study was processed according to the following data flow for the purpose of standardization and harmonization using imaging data analysis software Hermia and MIAKAT^29,30^. Raw DAT-SPECT projection data were imported into the Hermia and reconstructed using hybrid OSEM, with fixed iterations and subsets across all centres to ensure cross-site consistency, along with attenuation correction and post-filtering being omitted at the reconstruction stage by design^29^. The reconstructed volumes were subsequently processed in MIAKAT (v5.0), a validated quantitative neuroimaging toolbox for SPECT analysis with a defined pipeline, including normalisation, volume of interest (VOI) selection, and semi-quantification. Notably, a Chang zero-order attenuation correction (scanner-specific μ values) and Gaussian smoothing (6 mm full width at half maximum) were applied^30^. Each SPECT volume was affine-registered (12-parameter) to a dopaminergic SPECT template in the MNI152 space for spatial standardisation, and normalisation was visually quality-checked to exclude scans failing prespecified criteria^30^. The known anterior–posterior and dorsal–ventral gradients of the striatum were captured employing a 12-region subregional parcellation from the CIC hierarchy, including bilateral pre-/post-caudate and pre-/post-putamen compartments divided into dorsal and ventral zones^30^. Pre-/post-commissural divisions followed the anterior commissure, whereas dorsal–ventral subdivision followed atlas-based voxel-wise definitions with the posterior commissure serving as an anatomical landmark^31,32^. Finally, CIC Atlas-derived VOIs (via MIAKAT) were used to extract mean standardized uptake values in target striatal regions (SUV_TAR) and in the reference region (SUV_REF)^33–35^. Standardized uptake values (SUVs) were calculated by normalizing regional radioactivity concentrations to the injected dose and body weight. Following this, the SUV_REF was used to denote the supratentorial cerebral white matter, and SBRs were computed as follows: SBR_TAR = (SUV_TAR/SUV_REF)—1^31,32^.

### SuStaIn analysis

Herein, the SuStaIn algorithm, a probabilistic machine-learning framework that simultaneously identifies disease subtypes and reconstructs their progression trajectories from cross-sectional data, was applied^9^. The algorithm clustered individuals sharing similar temporal patterns of regional abnormality and inferred the sequential order in which these abnormalities emerge within each subtype. Analyses were performed using the z-score model implemented in pySuStaIn^36^, defining disease events as deviations of 1, 2, and 3 standard deviations from the HC means, with a maximum z-score of 5. DAT-SPECT data from 12 striatal regions were then included, with age, sex, site, and handedness entered as covariates to control for potential confounding effects. Models were tested with one to five subtypes using 200,000 Markov chain Monte Carlo iterations across 100 starting points. Model selection was based on the Bayesian Information Criterion^37^, and each participant was assigned a subtype and disease stage via maximum-likelihood estimation.

To assess temporal stability, the baseline-trained model was applied to follow-up DAT-SPECT scans acquired at 1-, 2-, and 4 years. All follow-up images underwent identical pre-processing using baseline-derived parameters to maintain cross-time consistency. Agreement between baseline and follow-up subtype assignments was quantified using Cohen’s kappa coefficient (*κ*)^38^.

As a sensitivity analysis, we assessed whether the identified subtypes were primarily driven by hemispheric laterality. We therefore performed an additional SuStaIn analysis after aliging hemispheric involvement according to the more affected posterior putamen (MAPP/LAPP framework). For each baseline Parkinson’s disease participant, we calculated hemisphere-specific posterior putamen SBR as the sum of dorsal and ventral posterior putamen SBRs; the hemisphere with the lower summed posterior putamen SBR was defined as the MAPP, and the contralateral hemisphere was defined as the LAPP. The 12 striatal regions were then reordered into a MAPP/LAPP-aligned framework prior to model fitting. Using this reordered dataset, we re-estimated the z-score SuStaIn model with the same covariates (age, sex, site, and handedness) and model-selection procedure as in the primary analysis. Concordance between the original S1, S2, and S3 assignments and the MAPP/LAPP-aligned subtype assignments was evaluated by cross-tabulation at baseline.

To examine whether a simplified representation of the striatum could capture similar subtype patterns, we repeated the SuStaIn analysis using a four-region parcellation (bilateral caudate and putamen) based on the PPMI quantitative DAT-SPECT dataset. The z-score SuStaIn model was re-estimated using the same modelling framework, covariates (age, sex, site, and handedness), and model-selection procedure as in the primary analysis.

### Statistical analyses

All statistical analyses were conducted in IBM SPSS Statistics (version 29.0; IBM Corp., Armonk, NY). Baseline group comparisons were performed using Kruskal–Wallis tests for four groups (HC, S1, S2, and S3) and three Parkinson’s disease subtypes (S1, S2, and S3). When omnibus tests were significant, pairwise post-hoc comparisons were performed using the Bonferroni adjustment (Table 1). Cross-sectional associations at baseline were examined using linear regression within each group (HC, S1, S2, and S3), modelling disease stage as the predictor and clinical measures as outcomes. Standardized β coefficients and *P* values are presented in Supplementary Table 1, with illustrative scatterplots and 95% confidence intervals (CI) shown in Supplementary Figs. 3 and 4. Primary longitudinal analyses of annualized change from baseline to 1 and 2 years were conducted using ANCOVA, with Subtype (S1/S2/S3) as the between-subject factor. Covariates included baseline outcome value, age, sex, years of education, imaging site, and LEDD at each visit. Estimated marginal means were reported with 95% CIs, omnibus F- and *P*-values for the Subtype effect, and partial η² as the effect size, with pairwise subtype contrasting Benjamini–Hochberg false discovery rate adjusted within outcome (q < 0.05) (Table 2). For baseline group analyses, Bonferroni correction was applied to control the family-wise error rate across multiple pairwise comparisons, whereas for longitudinal ANCOVA analyses, Benjamini–Hochberg false discovery rate correction was applied within each outcome to balance control of type I error with statistical power. All tests were two-sided, with statistical significance set at *P* < 0.05.

## Supplementary information

Supplementary Information

## Data Availability

The data analyzed in this study were obtained from the PPMI database (www.ppmi-info.org). PPMI is an open-access resource; qualified investigators can apply for access to the clinical, imaging, and biomarker data through the PPMI website. The authors had no privileged access. Processed DAT-SPECT quantification used in this work was centrally generated by the PPMI imaging core using the standardized HERMES/MIAKAT pipeline and was used as released, without local re-segmentation.
