# Supplementary material for: DAT-SPECT-based subtype and stage inference in Parkinson’s disease
Source: NPJ Parkinsons Dis. 2026 Apr 15;12:142. doi: 10.1038/s41531-026-01347-2 (PMC13265722; doi:10.1038/s41531-026-01347-2)

**Supplementary Figure 1. CONSORT-style flow diagram of cohort selection**

Legend. Consecutive PPMI participants at baseline (January 2011–December 2024) and stepwise exclusions: genetic PD, prodromal RBD/hyposmia, SNCA, SWEDD; then analysis-specific exclusions (non-zero LEDD at baseline, scans lacking 12-region parcellation, 99mTc-TRODAT-1 acquisitions). Final analytic cohorts: 126 healthy controls and 636 drug-naïve sporadic PD.


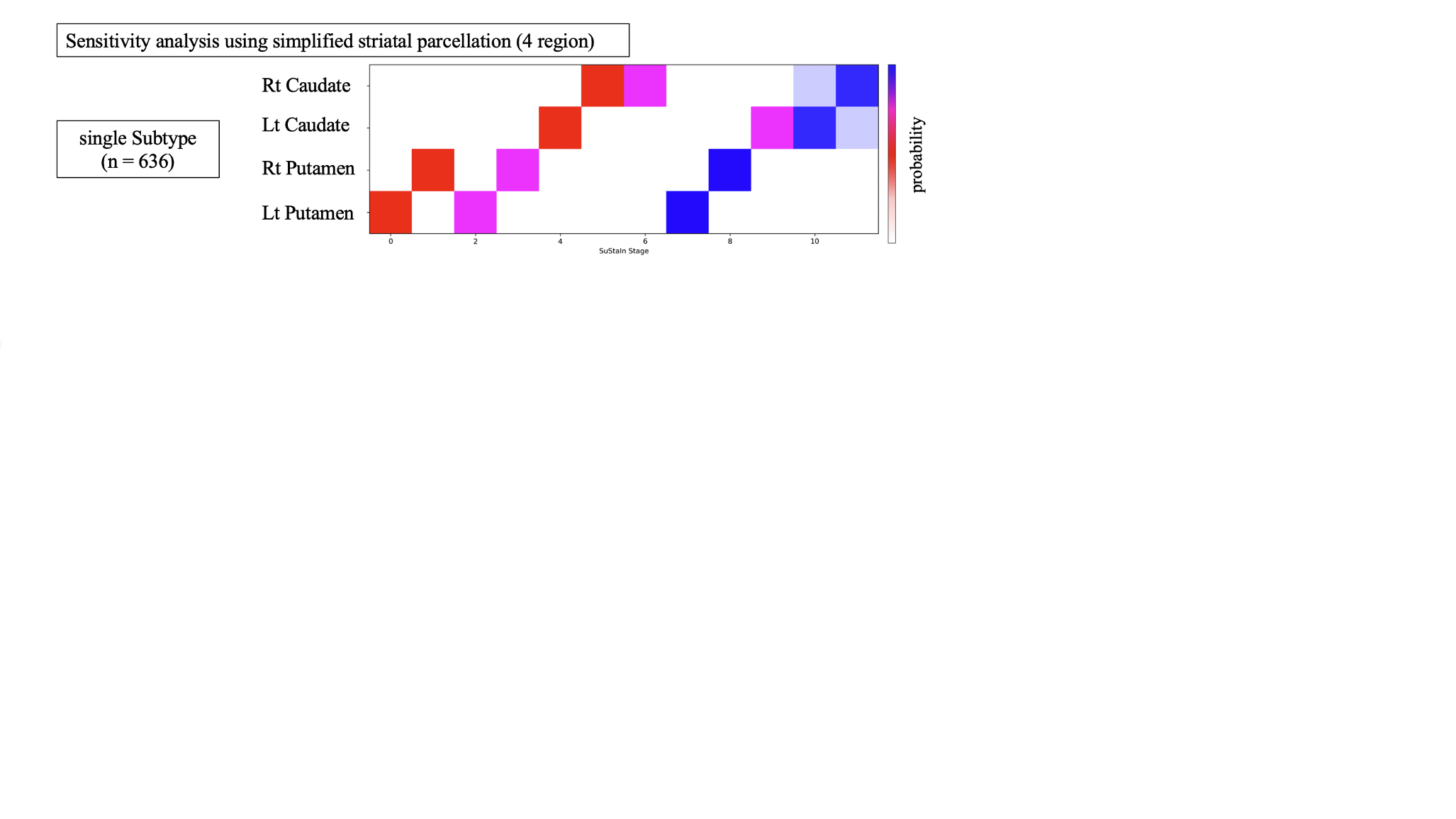


**Supplementary Figure 2. Sensitivity analysis using a simplified four-region striatal parcellation.**

Legend. The SuStaIn model was re-estimated using a simplified striatal representation comprising four regions (right and left caudate, right and left putamen). The model converged to a single-subtype solution in the drug-naïve Parkinson’s disease cohort (n = 636). The positional variance diagram shows the probabilistic ordering of dopaminergic signal reduction across SuStaIn stages. Compared with the primary 12-region analysis, the reduced regional resolution limited the model’s ability to resolve heterogeneous subtype trajectories.

**Supplementary Figure 3. Cross-sectional associations between SuStaIn Stage and clinical measures at baseline (set 1)**

Legend. Scatter plots with regression lines (and 95% CIs) stratified by subtype showing baseline associations of stage with: (A) Age, (B) MDS-UPDRS Part I, (C) MDS-UPDRS Part II, (D) MDS-UPDRS Part III, (E) UPSIT, (F) MoCA. Formal statistics are reported in Supplementary Table 1.

**Supplementary Figure 4. Cross-sectional associations between SuStaIn Stage and clinical measures at baseline (set 2)**

Legend. Additional scatter plots with regression lines for: (A) QUIP (ICD), (B) ESS, (C) RBDSQ, (D) GDS/BDI, (E) STAI, (F) SCOPA-AUT. Formal statistics are reported in Supplementary Table 1.


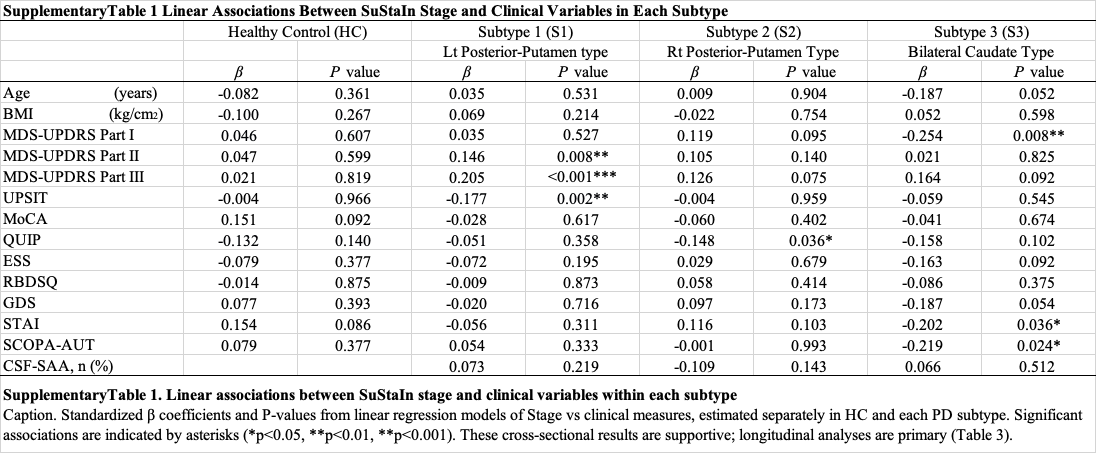

Supplement: Supplementary file 1 — Supplementary Information [file 41531_2026_1347_MOESM1_ESM.docx]
